# “There hasn’t been a push to identify patients in the emergency department”—Staff perspectives on automated identification of candidates for pre-exposure prophylaxis (PrEP): A qualitative study

**DOI:** 10.1371/journal.pone.0300540

**Published:** 2024-03-14

**Authors:** Samantha A. Devlin, Amy K. Johnson, Kimberly A. Stanford, Sadia Haider, Jessica P. Ridgway

**Affiliations:** 1 Department of Medicine, Section of Infectious Diseases and Global Health, University of Chicago, Chicago, Illinois, United States of America; 2 Division of Adolescent and Young Adult Medicine, Department of Pediatrics, Ann & Robert H. Lurie Children’s Hospital of Chicago, Chicago, Illinois, United States of America; 3 Feinberg School of Medicine, Northwestern University, Chicago, Illinois, United States of America; 4 Department of Medicine, Section of Emergency Medicine, University of Chicago, Chicago, Illinois, United States of America; 5 Department of Medicine, Section of Obstetrics and Gynecology, Rush University, Chicago, Illinois, United States of America; Beth Israel Deaconess Medical Center/Harvard Medical School, UNITED STATES

## Abstract

Automated algorithms for identifying potential pre-exposure prophylaxis (PrEP) candidates are effective among men, yet often fail to detect cisgender women (hereafter referred to as “women”) who would most benefit from PrEP. The emergency department (ED) is an opportune setting for implementing automated identification of PrEP candidates, but there are logistical and practical challenges at the individual, provider, and system level. In this study, we aimed to understand existing processes for identifying PrEP candidates and to explore determinants for incorporating automated identification of PrEP candidates within the ED, with specific considerations for ciswomen, through a focus group and individual interviews with ED staff. From May to July 2021, we conducted semi-structured qualitative interviews with 4 physicians and a focus group with 4 patient advocates working in a high-volume ED in Chicago. Transcripts were coded using Dedoose software and analyzed for common themes. In our exploratory study, we found three major themes: 1) Limited PrEP knowledge among ED staff, particularly regarding its use in women; 2) The ED does not have a standardized process for assessing HIV risk; and 3) Perspectives on and barriers/facilitators to utilizing an automated algorithm for identifying ideal PrEP candidates. Overall, ED staff had minimal understanding of the need for PrEP among women. However, participants recognized the utility of an electronic medical record (EMR)-based automated algorithm to identify PrEP candidates in the ED. Facilitators to an automated algorithm included organizational support/staff buy-in, patient trust, and dedicated support staff for follow-up/referral to PrEP care. Barriers reported by participants included time constraints, hesitancy among providers to prescribe PrEP due to follow-up concerns, and potential biases or oversight resulting from missing or inaccurate information within the EMR. Further research is needed to determine the feasibility and acceptability of an EMR-based predictive HIV risk algorithm within the ED setting.

## Introduction

Pre-exposure prophylaxis (PrEP) is a safe and highly effective form of HIV prevention; however, there are significant disparities in PrEP coverage based on race and ethnicity, age, and sex. Black and Hispanic/Latino individuals account for the majority of people for whom PrEP is recommended yet have the lowest rates of PrEP use among all racial and ethnic groups [1]. Additionally, PrEP coverage was about three times as high in 2020 among males (28%) as among females (10%) [1]. The low rate of PrEP use among cisgender women (hereafter referred to as “women”) is particularly concerning because they accounted for 19% of all new HIV diagnoses in the United States in 2019, demonstrating a critical imbalance in PrEP coverage that should be addressed [1].

The emergency department (ED) is an apt setting for leveraging the electronic medical record (EMR) system to utilize algorithms for identifying candidates for PrEP. Patients who access the ED often have co-occurring social and health risk factors for potential exposure to HIV [2–9]. Targeted screening demonstrates that many ED patients are considered at risk for HIV and prime candidates for PrEP based on their self-reported behaviors [10]. However, logistical factors complicate prioritizing HIV risk screening and access to PrEP services [11, 12]. ED staff have minimal time for HIV risk screening, and for those who do practice routine HIV testing, it is difficult to perform HIV prevention counseling and risk assessment for all patients who test negative [13, 14].

Predictive algorithms can help to minimize the time required for conducting HIV risk screening among patients in the ED. For example, an automated system can be developed in which an electronic risk score is automatically calculated at triage for each patient. The algorithm would utilize information within the EMR such as age, gender, gender of sexual partner, chief complaint, and positive test for a sexually transmitted infection (STI) in the prior 6 months and would send an alert to an HIV prevention counselor for patients whose risk score is above a given threshold. This counselor would then perform real-time HIV prevention counseling, risk assessment, and PrEP linkage as appropriate [15]. These types of algorithms have effectively identified men who are at risk for HIV and have led to appropriate PrEP referral and prescription; however, such algorithms often have lower sensitivity for identifying women who are vulnerable to HIV and in need of PrEP, as women’s risk factors for HIV acquisition may not be documented in the EMR; this disparity may contribute to the vast under-prescription of PrEP to women compared to men in the U.S. [15–19].

Despite challenges associated with implementing automated identification of PrEP candidates, the ED can serve as an access point for PrEP referral and can reach underserved populations at increased risk for HIV who may not otherwise engage in outpatient care [7]. Furthermore, patients within the ED are increasingly interested in initiating PrEP [6]. In combination with other efforts to improve PrEP uptake, offering PrEP referral in the ED could help to increase PrEP utilization and avert HIV infections. This exploratory study aimed to a) understand the perspectives of ED staff regarding existing processes for identifying patients who are at risk for HIV and ideal candidates for PrEP and b) explore the facilitators and barriers of implementing automated identification of PrEP candidates within the ED, with specific considerations for women.

## Materials and methods

### Design

We used a content analysis approach to conduct a focus group with four patient advocates and four semi-structured qualitative interviews with physicians who worked in the University of Chicago Medicine Emergency Department (UCM ED), a high-volume urban tertiary care hospital ED with a level 1 trauma center and HIV screening program. Participants were eligible if they either worked full-time or part-time in the UCM ED, were aged 18 years or older, and able to speak and understand English and to provide informed consent. Staff were recruited from March to July 2021 using snowball sampling, as well as purposive sampling via emails to specific hospital-based listservs that included resident and attending physicians, nurses, and patient advocates in the ED. Staff members who were interested in participating in the study contacted the research team, and a clinical research coordinator confirmed their eligibility and scheduled a time to conduct the interview. Ultimately, 8 ED staff members were enrolled in the study. No participants dropped out of the study.

All interviews were conducted remotely over Zoom or phone by a clinical research coordinator (SAD; MS degree) or a research associate professor (AKJ; PhD, MSW degrees) in a private, secure location from May to July 2021. Both the research coordinator and the research associate professor were female and had extensive experience in qualitative research data collection and analysis on HIV risk/prevention and PrEP, particularly among women. Prior to study commencement, all participants were made aware of the interviewers’ reasons for conducting the research and their interest in improving PrEP awareness and uptake among women.

Interviews lasted approximately 30 to 60 minutes. The focus group was conducted over Zoom by the research associate professor and lasted 60 minutes. Participants provided informed verbal consent, which was audio recorded and witnessed by the research coordinator and/or research professor. As the study posed no more than minimal risk to participants, verbal consent was approved by the relevant Institutional Review Boards (IRBs). Participants could skip questions or stop the discussion at any time. All discussions were audio recorded and professionally transcribed. Interviewers had access to information that could identify individual participants during and after data collection. However, no personally identifiable information (e.g., name) was collected on the audio recording. Field notes were taken during interviews and the focus group. Participants received $15 through an electronic payment application. This study was approved by the IRBs at the University of Chicago (IRB19-1345) and Ann & Robert H. Lurie Children’s Hospital of Chicago (IRB 2021–4506).

The semi-structured interview guide was designed to gather information about ED staff’s PrEP knowledge; existing processes for identifying patients at risk for HIV and for prescribing PrEP; and thoughts on utilizing an automated algorithm for identifying PrEP candidates within the ED (Table 1).

**Table 1:** Example questions from interview/focus group discussion guide.

Broad domain of qualitative exploration	Sample exploratory qualitative questions	Themes elicited from qualitative data
Emergency department and knowledge of PrEP/HIV prevention	• Thinking about patients who come to the ED, do you think PrEP is something that they should be aware of? • Have you completed any PrEP-related training? If not, would you be interested in PrEP educational training? • How would you describe your comfort level in discussing HIV risk factors and sexual health topics with patients in the ED? Is your comfort level in discussing these topics the same for men and women?	1. Major theme: Limited PrEP knowledge among ED staff, particularly regarding its use in women a. PrEP is within the scope of emergency medicine b. Limited to no PrEP training among ED staff c. Limited knowledge of PrEP guidelines/considerations for women
Existing processes in the ED for HIV risk assessment/PrEP screening	• In the ED, what is the process for determining if someone is vulnerable to HIV and eligible for PrEP? • What are some concerns/barriers in regard to prescribing PrEP within the ED?	2. Major theme: The ED does not have a standardized process for assessing HIV risk and identifying ideal PrEP candidates a. No standardized process for assessing patients’ HIV risk/need for PrEP b. Continuity of care and cost/insurance concerns related to PrEP referral or prescription c. Opportunity for identifying people most vulnerable to HIV within the ED
Implementing a refined automated identification process in the ED	• Why should an automated system exist, or why should an automated system not exist specifically within the ED? • What kind of department support and training would you need to implement a refined automated identification process for HIV/PrEP screening in the ED? • Who would need to be involved and what resources would be required to ensure successful implementation?	3. Major theme: Perspectives on and barriers and facilitators to an automated algorithm for identifying ideal PrEP candidates a. Perspectives: Automated screening would identify a large number of people b. Barriers: An automated algorithm could overlook people who are at high risk for HIV if relevant data are not reported/documented within the EMR c. Facilitators: Organizational buy-in, patient and staff support, and EMR optimization would be required for implementation

### Rigor, validity, and reliability

During data collection and analysis, the two interviewers met routinely to review and summarize preliminary findings while making iterative adjustments to ensure the principal research questions were being adequately addressed and to assess data saturation. Researcher reflexivity was performed during both data collection and analysis, with the goal of highlighting participants’ viewpoints without any interference from our research team’s subjective interpretations or biases. Transcripts were examined using deductive thematic content analysis via Dedoose, an online qualitative and mixed methods research software [20].

A preliminary codebook was developed from the interview guide with clear definitions for each code. The primary and secondary coder (i.e., two interviewers) reviewed and revised the preliminary codes. Codes were applied to all five transcripts (4 individual interviews; 1 focus group) by the primary coder and were reviewed by the secondary coder for consensus. Most divergences occurred due to omission, and upon review and revision by both coders, were quickly rectified to 100% agreement. Codes were then applied to all transcripts and were reviewed by all team members (two interviewers and principal investigator) for consensus. Themes were derived based on code prevalence within Dedoose. Each theme was distinct and had enough supporting data to be considered significant. Saturation was determined when there was a high prevalence of code clustering and no emergence of new themes. The primary coder classified the major themes found across transcripts and presented the findings to the research team for final consensus [21]. Relevant quotations were selected to highlight significant themes. We invited participants to review the paper, one of whom (ED physician) provided feedback (e.g., context/important information regarding the ED setting and typical roles/responsibilities of staff members) that was incorporated prior to submission.

## Results

From May to July 2021, we conducted 1 focus group with 4 patient advocates (PtAdv) and 4 individual interviews with ED physicians (EDP#). Most participants identified as Black/African American (4, 50.0%) and cisgender female (5, 62.5%) (Table 2). We found three major themes across interviews related to current processes for identifying PrEP candidates and potential barriers and facilitators of an automated algorithm for identification of patients who are most vulnerable to HIV and could benefit from PrEP.

**Table 2:** Participant demographics (N = 8).

Category	Total
	n (%)
**Age**	
18–29	3 (37.5)
30–39	2 (25.0)
40–49	2 (25.0)
50–59	1 (12.5)
60 or older	0 (0.0)
**Race** ^**a**^	
Black/African American	4 (50.0)
White	3 (37.5)
Unknown	1 (12.5)
**Gender** ^**b**^	
Cisgender female	5 (62.5)
Cisgender male	2 (25.0)
Other	1 (12.5)
**Ethnicity**	
Hispanic/Latino	0 (0.0)
Not Hispanic/Latino	8 (100)
**Job Role**	
Patient advocate/non-clinical staff	4 (50.0)
ED resident physician	3 (37.5)
ED attending physician	1 (12.5)
**Time Spent in Current Job Role**	
Median	2 years
Range	7 months– 5 years

^a^Black/African American, White, Asian/Asian American/Pacific Islander, Middle Eastern/Arab American, Native American/American Indian/Alaska Native/Indigenous, and Unknown

^b^Cisgender female, Cisgender male, Transgender female, Transgender male, Non-binary/queer, and Other

### 1) Limited PrEP knowledge among ED staff, particularly regarding its use in women

There was minimal knowledge about PrEP among participants. One ED physician confused PrEP with post-exposure prophylaxis (PEP).

I was thinking about PEP earlier and now it’s about PrEP…maybe we do have PrEP in the ED. I don’t even know if we do or not, so…that’s something I need to find out. (EDP3)

A patient advocate stated that their knowledge of PrEP was likely constituted of misconceptions, which could have resulted from a lack of training.

I didn’t get any formal trainings on PrEP. And I think most of what I know now is probably myths about PrEP, right? But I don’t–I can’t even tell you if they’re true or false… (PtAdv)

In particular, physicians and patient advocates alike discussed how they “have not thought a lot about PrEP in heterosexual females” (EDP1).

I know its [PrEP]…basic function is to prevent HIV transmission…I don’t know a whole lot about its use specifically in women. I associate it more with gay men. So, I, to be honest, don’t know a whole lot [about PrEP for women]. (EDP2)

I don’t think I even realized until a couple of years ago that it [PrEP] was even something that they would say–that they were even opening up to women. I think I really just found out maybe a couple of months ago that Truvada was the same thing [Truvada is a form of PrEP]. (PtAdv)

This lack of knowledge about PrEP among women was particularly concerning due to the typical population seen in the ED.

We see females more often coming in with complaints related to STDs [sexually transmitted diseases]…for example, you know, vaginal discharge is something we see all the time in the ER, way more than we see something like, you know, dysuria in a male…I see…many more female patients. (EDP1)

### 2) The ED does not have a standardized processes for assessing HIV risk and identifying ideal PrEP candidates

Physicians reported an absence of a standardized process for assessing a patient’s HIV risk and need for PrEP. Most of the time, providers use the patient’s presenting complaint or common HIV risk factors identified as part of clinical care, in addition to their own “backgrounds and preexisting [PrEP] knowledge,” (EDP2) when determining if a patient is a good candidate for PrEP.

It’s basically, if you think they might be high risk based on why they’re there in the ED, you say to them, ‘Have you ever heard of it [PrEP] and if you’re interested, here’s where you get some more information,’ but no, we don’t have anything standardized. (EDP4)

One physician stated that “there’s no way for me to know for sure which patients are and are not potentially at risk [for HIV]” (EDP2) and wanted “a reminder that’s even like a thing [PrEP]…like, which patients are eligible” (EDP2).

I think really what is important for us to know is just what it [PrEP] is, who would be a good candidate for it, and how do we get to it essentially, and all the rest of the questions, generally, we defer to the person prescribing it just because it’s a limited amount of time we have in the ED to address this. (ED4)

Providers agreed that it is important for them to assess someone for PrEP eligibility but voiced that they should not be the ones responsible for actually prescribing PrEP or facilitating subsequent follow-up care.

I think that on a national level people have had this discussion about PrEP in the emergency department and the kind of general feeling is that PrEP should not be prescribed from the emergency department. And…that if we identify someone who we think is eligible for PrEP, we should be referring them somewhere else for that to be done. And so, I think because of that, there hasn’t been a push to identify patients in the emergency department [who could benefit from PrEP]. (EDP1)

Despite reporting that PrEP should not be prescribed from the ED, patient advocates and physicians alike believed that the ED is “the perfect place to introduce” something like PrEP (PtAdv) because they serve a “higher risk population” (EDP3).

When the community comes into the emergency room, they’re at a point where they’re looking for something…And so, I believe that this is probably one of the best places to get them to say ‘Yes’, especially when it comes to consideration for their health or consideration for testing, because a lot of times, when we talk about access, this is the only place they know to get access. (PtAdv)

However, physicians specifically mentioned how their limited time often prevents them from discussing sexual health topics and performing HIV risk assessment within the ED, unless “somebody is there for a complaint that is related to something that might be suspicious for an STI” (EDP4).

Oh, I think it’s [PrEP] 100% in the scope of emergency medicine and I wish that we did it more [discuss HIV risk and PrEP]…But it comes down to, you know, I may only have a few minutes with that patient, and I have 15 other things to do in the ER. (EDP1)

### 3) Perspectives on and barriers/facilitators to an automated algorithm for identifying ideal PrEP candidates within the ED

Participants were asked about their perspectives on utilizing an EMR-based automated algorithm for identifying people who are most vulnerable to HIV and could benefit from PrEP.

Physicians stated that such an algorithm would be a good idea as “automated processes work well” (EDP2) and it “could be useful…we would be responsive to [it] because we’re used to [them] already” (EDP1) and thought it would work best if the algorithm was based “on a complaint or if a patient discloses something in history that could trigger it” (EDP1).

I think…if there’s a way to link it back to their chart or even someone who has been tested for gonorrhea or chlamydia in the last six months…[or] multiple times in the last several months…that would be another flag…I think that’s a great way to identify them [ideal PrEP candidates]. (EDP1).

Although there was acceptability of an EMR-based algorithm among some participants, others raised concerns and potential barriers to utilization. Two physicians thought the algorithm may be inaccurate if it was based on data from the EMR because it may inadvertently exclude certain patients who may need PrEP but do not have “glaringly obvious [risk factors] in their chart” (EDP3), creating biases.

Most of the PrEP eligibility criteria, at least as of what we can identify in the ED, it’s all diagnosis with another STI…You can’t automate it if it’s not in the chart and we never ask people about sexual behaviors or risk behaviors unless they’re there with something that would prompt those questions. I feel like it would be tough to identify anybody other than the people who are there with the complaints that are already addressed off of PrEP. (EDP4)

Additionally, patient advocates thought that the EMR-based algorithm would “make it easier to identify [people at risk for HIV and in need of PrEP]” but it may not be widely accepted among some patients, stating that “the patient’s willingness to want to do it…[is] gonna be the main barrier” (PtAdv).

“That’s [an EMR-based algorithm] a form of tracking…And there are a lot of people who do not want to be identified in terms of those ways…in that regard, there will be some pushback about that. And…especially in communities where the trust level is not there. They come to the emergency room because they don’t want to commit to having someone know about their full medical record. So, if you’re introducing something that has a tracking mechanism attached, I think you will get some pushback about that.” (PtAdv)

To combat this barrier, patient advocates stressed the importance of patient buy-in for using such an algorithm.

It’s just about getting the patient to buy in, so you just really want to make sure that they understand…the benefit to them, not so much about the tracking and that people are able to get into this information and hack this and hack that. But it’s really just assuring them that this is the best way to move forward with whatever part of their life that they’re at.” (PtAdv)

Likewise, physicians stated that an EMR-based automated algorithm for identifying PrEP candidates would require organizational support, staff buy-in, and optimizing the EMR.

The biggest thing is the buy-in. I think training also. I think it’s gonna really depend on how easy the process is and how much time it will add to…the workflow of the nurses and the residents…I would think that it’s a great idea to try. (EDP2)

## Discussion

To our knowledge, this is the first study to examine ED staff perspectives on using an automated algorithm for assessing HIV risk and identifying potential candidates for PrEP. Despite limited knowledge of PrEP, particularly for women, and no standardized protocol for HIV risk assessment and PrEP referral within the ED, participants believed that PrEP should be considered within the scope of emergency medicine [22]. Our physicians’ concerns around prescribing PrEP, including continuity of care, time constraints, and competing priorities have been voiced by providers in other studies [23–27]. To increase provider comfort regarding PrEP screening, education, and referral, it is important for organizations to make them aware of local care referral information and the latest PrEP guidelines to better understand which individuals are most vulnerable to HIV and to link them to the appropriate services [22].

Participants expressed limited knowledge about PrEP, particularly regarding its use among women. This lack of knowledge has important implications for the use of future automated algorithms for identifying PrEP candidates within the ED. If providers do not feel comfortable discussing PrEP with women who are vulnerable to HIV, then they are unlikely to utilize an automated algorithm or act upon the results either by prescribing PrEP or referring women to PrEP providers. Moreover, if providers are more comfortable discussing algorithm results and PrEP with men than women, this could exacerbate disparities in PrEP use based on sex, as women are already disproportionately underrepresented among PrEP users.

The idea of an EMR-based algorithm for assessing HIV risk and identifying PrEP candidates within the ED was supported by our participants. Indeed, an automated HIV risk model in which patients receive PrEP education and local referral information has been shown to be feasible within the ED setting [28] and may also improve adherence to best practices in HIV prevention within the ED [29, 30]. Our participants highlighted both potential barriers and facilitators of an automated algorithm for identifying candidates who could benefit from PrEP. Patient advocates were concerned that there would be distrust of the algorithm and low acceptability among patients who typically use the ED and do not have an established relationship with one provider, whereas providers raised the issue of potential biases or oversights that could result from missing or inaccurate data within the EMR. For example, ED providers may not obtain a sexual history for all patients, and so behaviors such as condomless sex with multiple partners may not be documented in the EMR. Most EMRs do not have structured fields to capture some of the nuanced behavioral factors that have been associated with HIV acquisition among women, such as the gender of a woman’s sexual partner’s other partners. Concerns about missing EMR data were also voiced by primary care providers in a similar study [31]. It is important that ED providers are trained on how to accurately take a social and sexual history from patients so that data within the EMR are accurate and complete.

Finally, our participants reported that utilizing an EMR-based algorithm would require buy-in from patients and staff alike. Organizational support and potential updates to the EMR (e.g., adding structured fields for behavioral factors associated with HIV acquisition) or the ED workflow may also be required to ensure that the algorithm is running properly and being correctly interpreted by staff. Additionally, ensuring that providers (e.g., nurses, physicians, etc.) are trained on documenting social and sexual histories within the EMR will be crucial, as the algorithm would rely on data within the EMR. In particular, understanding different HIV risk factors for women will be important for accurately identifying their risk compared to men [15, 16, 32]. Due to the time constraints imposed on ED providers, it is especially important that risk factors are accurately documented within the EMR for patients with STI-related complaints. Although the ED physicians in our study did not believe that they should prescribe PrEP, it is important that ED providers have access to local referral information. Additionally, it would be practical to continually evaluate what ongoing support is required to make sure that individuals who could most benefit from PrEP are identified within the ED and linked to the appropriate services [7].

There are a few limitations to our study. The sample size was small due to the impact of COVID-19 on our ability to recruit ED staff who were burdened by competing demands. Although the sample size was relatively small, our data adequacy still allowed us to reach data saturation, which can be achieved in a limited range of interviews/focus groups, particularly in health research studies with relatively homogeneous populations and narrowly defined objectives [33–38]. Our results are framed in the context of an exploratory study for this specific ED setting and may not transfer to smaller or rural healthcare centers or EDs without robust HIV screening programs. Future studies should expand the sample size and interview staff across multiple roles within the ED, particularly nurses, who would play a crucial role in facilitating HIV risk assessments and identification of PrEP candidates within the ED. Additionally, the concerns raised by ED staff regarding patient trust of an EMR-based algorithm also highlight the need to conduct a similar exploratory study among patients who access the ED.

## Conclusions

This exploratory study is a first step toward assessing the acceptability and feasibility among ED staff of utilizing automated algorithms for HIV risk assessment. Participants recognized the utility and associated challenges of identifying potential PrEP candidates within the ED via an EMR-based automated algorithm. Improving PrEP awareness and referral within the ED may help to reach individuals who are not regularly engaged in the healthcare system and those who are most vulnerable to HIV, particularly among women who are under-prescribed PrEP according to their need. Future studies should include patients who access the ED and expand the number of ED staff to determine additional facilitators/barriers to implementing an EMR-based automated algorithm for identifying ideal PrEP candidates within the ED.
